# Can Eucalyptol Replace Antibiotics?

**DOI:** 10.3390/molecules26164933

**Published:** 2021-08-14

**Authors:** Wanda Mączka, Anna Duda-Madej, Aleksandra Górny, Małgorzata Grabarczyk, Katarzyna Wińska

**Affiliations:** 1 Department of Chemistry, Wroclaw University of Environmental and Life Sciences, Norwida 25, 50-375 Wrocław, Poland; aleksandra2.gorny@gmail.com; 2 Department of Microbiology, Wroclaw Medical University, Chałubińskiego 4, 50-368 Wrocław, Poland; anna.duda-madej@umed.wroc.pl

**Keywords:** eucalyptol, 1,8-cineole, antimicrobial activity

## Abstract

One of the primary reasons for the search for new antimicrobial agents is the increasing and spreading resistance of microorganisms to previously used drugs. This is particularly important in the case of rapidly progressing infections that require the rapid administration of an appropriately selected antibiotic. However, along with the administration of antibiotics, complications in the disease-weakened body may arise in the form of systemic mycoses, viral infections, and protozoan infections. Therefore, there is an increasing interest among researchers focusing on the use of naturally occurring terpenic compounds in stand-alone or combined therapies with antibiotics. In this publication, the aim of our work is to present the results of a literature review on the antimicrobial activity of eucalyptol.

## 1. Introduction

Preparations based on ingredients of natural origin seem to be an excellent alternative to synthetic drugs in the prevention and treatment of many diseases, including infectious diseases. This is due to their comprehensive action on the human body while generating a relatively small number of side effects [1,2,3].

An additional advantage of natural antimicrobial compounds is the lower probability of microbial resistance, unlike traditional antibiotic therapy [4]. Thus, the question arises of whether simple compounds of natural origin can be used as a substitute for traditionally used drugs. Will they prove to be sufficiently effective? In this publication, we would like to present the results of the literature review on the antimicrobial properties of the cyclic terpenoid ether: eucalyptol (1,8-cineole).

**Figure 1:**
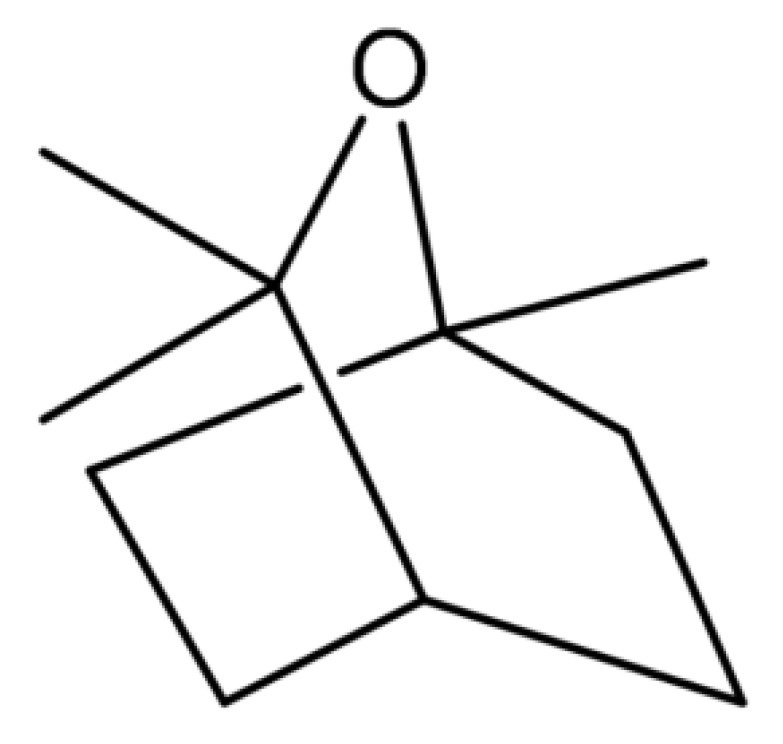
Structure of 1,8-cineole.

1,8-Cineole (1,3,3-trimethyl-2-oxabicyclo[2.2.2]acetate) (Figure 1), also known as eucalyptol, is a bicyclic terpenoid that is a component of essential oils obtained from various plant species. It is the main ingredient (30–90% content) of essential oils obtained from eucalyptus leaves, e.g., *Eucalyptus smithii*, *E. globulus* Labill, *E. maidenii*, *E. bicostata*, *E. sideroxylon*, *E. cinerea* and *E. leucoxylon* [5]. Essential oil of eucalyptus used for medicinal purposes should contain at least 70 percent eucalyptol, according to the European and British Pharmacopoeia [6].

Eucalyptol is also an important component of essential oils obtained from other plants, such as tea tree, mugwort, rosemary, sage, niuola, kajeput, yarrow, and cinnamon, significantly affecting their properties and application [7,8,9].

Table 1 contains an overview of plants with a 1,8-cineole content of more than 60% in the volatile fraction.

## 2. Antimicrobial Activity

For research on the antibacterial activity of cineole, essential oils isolated from the leaves of various eucalyptus cultivars, in which a significant amount of this compound is present, as well as commercially available pure cineole, are used.

The essential oil was isolated from *Eucalyptus alba* growing in Senegal using the hydrodistillation method. In this EO, 18 ingredients were identified, constituting from 99.0 to 99.7% of the content. The main component was 1,8-cineole, with a content of 76.5–88.1%. Other ingredients of the essential oil were: limonene (3.8–8.6%), α-terpineol (1.4–2.8%), globulol (1.3–6.3%), and α-pinene (1.5–1.8%). The essential oil was tested for antibacterial properties against *Escherichia coli* ATCC 25922, *Enterococcus faecalis* ATCC 29212, *Staphylococcus aureus* ATCC 29213, and *Pseudomonas aeruginosa* ATCC 27853 using the disk method. Chloramphenicol was used as a positive control. The essential oil with a high content of 1,8-cineole (>76.5%) showed strong antibacterial activity against the *S. aureus* strain, good activity against *E. coli* and *E. faecalis*, and moderate activity against *P. aeruginosa*. The MIC values for the individual bacterial strains were also determined. They were 1.25 mg/mL (*S. aureus*), 6.25 mg/mL (*E. coli* and *E. faecalis*), and 25 mg/mL (*P. aeruginosa*), respectively [14].

In the essential oils of *Eucalyptus bicostata*, *E. cinerea*, and *E. sideroxylon*, found in Tunisia, 1,8-cineole (68.0–70.7%) was identified as the main component. The antimicrobial activity tests were performed against *E. coli* ATCC 25922, *P. aeruginosa* ATCC 27853, *S. aureus* ATCC 25923, and *E. faecalis* ATCC 29212 using the disc method. It was found that essential oils from *E. cinerea* and *E. sideroxylon*, containing α-terpineol (10.7% and 5.3%) and β-pinene (4.7% and 6.7%) in addition to 1,8-cineole (70.7% and 69.3%, respectively), showed less activity against *E. faecalis* and *S. aureus* than *E. bicostata* essential oil, which, in addition to 1,8-cineole (68.0%) and β-pinene (3.7%), also contained higher amounts of trans-pinocarveol (4.7%) and globulol (5.3%). It can be assumed that, due to their synergistic effects, these components could also contribute to a significant difference in activity between them [32].

Essential oils from the west Australian species of eucalyptus, *E. mallee*: *E. loxophleba*, *E. polybractea*, and *E. kochii* subsp. *plenissima* and subsp. *borealis*, were obtained by leaf hydrodistillation. The main component of all essential oils was 1,8-cineole, in the amount of 97.32% for *E. kochii* subsp. *borealis*, 96.55% for *E. kochii* subsp. *plenissima*, 82.95% for *E. polybractea*, 78.78% for *E. loxophleba* 2, 77.02% for *E. globulus*, and 66.93% for *E. loxophleba* 1. The essential oils were tested on a number of microorganisms, including Gram-positive bacteria: *S. aureus* ATCC 29213, methicillin-resistant *S. aureus* NCTC 10442, *E. faecalis* ATCC 29212, vancomycin-resistant *E. faecalis* ATCC 51299, and *Staphylococcus epidermidis* NCTC 11047; Gram-negative bacteria: *Salmonella enterica* subsp. *enterica* serovar Typhimurium ATCC 13311, *E. coli* ATCC 25922, *P. aeruginosa* ATCC 27853, and *Acinetobacter baumannii* NCTC 7844, and the yeast *Candida albicans* ATCC 90028. The essential oils inhibited the growth of the tested microorganisms to various degrees, with MIC values ranging from 0.25% to 8.0% (*v*/*v*). Essential oils obtained from *E. globulus* and *E. polybractea* showed the highest activity. With regard to the microorganisms, *E. faecalis* and *C. albicans* were the least resistant strains, whereas *A. baumannii* was the most sensitive [10].

Commercially available 1,8-cineole and the known chlorhexydine gluconate, were used for the next studies on inhibiting the growth of bacteria *S. aureus* ATCC 25923, methicillin-resistant *S. aureus*, *P. aeruginosa* ATCC 27853, *E. coli* ATCC 25922, *E. faecalis* ATCC 51299, and *Klebsiella pneumoniae* ATCC 700603, and yeast *C. albicans* ATCC 90028. For both compounds, their antimicrobial activity and their interactions were determined using a checkerboard analysis. Interactions between 1,8-cineole and chlorhexydrine gluconate have been described as synergistic, neutral, or antagonistic. A strong synergistic effect was observed with regard to *S. aureus* and methicillin-resistant *S. aureus* strains. For *E. coli*, *K. pneumoniae*, *E. faecalis*, and *C. albicans* strains, synergism was also observed, but was weaker. In the case of *P. aeruginosa*, the interaction was considered to be neutral [55].

Eucalyptol, amoxicillin/clavulanic acid (AMC), and gentamicin were tested *in vitro*, either alone or in combination, on three strains of *S. aureus* that were isolated from osteomyelitis patients. For all three strains, a synergistic effect of the combination of AMC with 1,8-cineole was observed, whereas the combination of AMC with gentamicin did not produce such an effect. In addition, the efficacy of these three antibacterial agents was tested in vivo using an experimental model of methicillin-resistant *S. aureus* that causes osteomyelitis in rabbits. The effectiveness of the therapy was assessed after four days of treatment by counting the number of bacteria in the bone marrow. A significant reduction in the number of bone marrow colonies was observed when rabbits were administered AMC with 1,8-cineole. Eucalyptol showed synergistic effects in combination with both AMC and gentamicin [56].

In the search for new agents acting on antibiotic-resistant strains of bacteria, 20 strains of methicillin-resistant *S. aureus* (MRSA) were tested for their ability to create biofilm. Then, the influence of the inhibition of the biofilm formation was examined by the action of the essential oil of *E. globulus* and its main component, which is 1,8-cineole. The study showed that 67.8% of the strains tested were sensitive to both the essential oil and 1,8-cineol. Biofilm development was inhibited by 74% to 91%, with both substances being active against most isolates at a concentration of 0.048 mg/mL [57].

The activity of 1,8-cineole towards specific microorganisms depends on its % share in individual plants. Table 2 shows the information collected on this topic.

*Melaleuca alternifolia* (tea tree) essential oil contains, among others, terpinen-4-ol and 1,8-cineole. The essential oil and its individual components have been tested as an antifungal agent against *Botrytis cinerea*. The results showed that 1,8-cineole significantly enhances the antifungal activity of terpinen-4-ol. Moreover, the antifungal activity of the two components together exceeds that of each of them individually, as well as the antifungal activity of the essential oil itself. Since terpinen-4-ol and 1,8-cineole are the two distinctive components of the essential oil and are clearly synergistic, their ability to damage membranes or intracellular components was investigated. Terpinen-4-ol has been found to be capable of destroying membrane integrity and increasing permeability, leading to ion leakage and membrane dysfunction. In turn, 1,8-cineole can penetrate the cell membrane and damage cell organelles without causing changes in the membrane [58].

The essential oil isolated from the leaves of *E. globulus*, found in Mexico, was tested for activity against strains of *B. cinerea* and *Colletotrichum acutatum*, which cause rotting diseases of grapes. The essential oil containing 68.26% of 1,8-cineole showed antifungal activity in vitro against both pathogens. The complete inhibition of *B. cinerea* growth was observed at a concentration of 3 µL/mL, whereas 2 µL/mL caused significant growth inhibition. In the case of *C. acutatum*, the use of 6 μL/mL and 4 and 5 μL/mL, respectively, was required to achieve similar results. *E. globulus* oil completely inhibited the germination of *B. cinerea* and *C. acutatum* at a concentration of 3 and 2 μL/mL, respectively [59].

Commercially available 1,8-cineole was used for studies to inhibit both the growth of *Aspergillus flavus* ATCC 22546 and the production of aflatoxins by this fungus. Eucalyptol was capable of a 50% inhibition of fungal growth at a concentration of 250 ppm. It was also characterized by a 50% inhibition of the production of aflatoxin B1 and aflatoxin B2 at a concentration of 100 ppm. These anti-flatoxygenic effects were associated with a drastic reduction in *aflE* and *aflL* expression by the test compound. The *aflE* and *aflL* genes are responsible for the expression of norsolorinic acid reductase and cytochrome P450 monooxygenase/desaturase. Norsolorinic acid reductase (NOR) is responsible for the conversion of NOR to averantin. This enzyme is involved in the biosynthesis of aflatoxins at a very early stage in the biosynthetic pathway. Furthermore, 1,8-cineole lowers the expression of the *aflL* gene, which encodes cytochrome P450 monooxygenase/desaturase and is believed to be involved in the conversion of versicolorin B (VERB) to VERA. This enzyme plays a key role in the separation of the AFB1 subtype from the AFB2 subtype. As a result of the action of 1,8-cineole, the biosynthesis of AFB1 will be blocked, and the biosynthesis of AFB2 will be enhanced [60].

## 3. Mechanism of Antimicrobial Activity

Quorum sensing (QS) is a system of communication between microbes by chemical signals, tightly controlled by genes that promote invasion, defense, and spread in response to the size of the bacterial population. It plays an important role in the process of the formation and functioning of biofilm, i.e., an organized microworld that is designed to protect the microorganisms that form them against the harmful effects of the external environment. Both of these processes are of great importance in the physiology of the bacterial cell, e.g., in the virulence of pathogenic bacteria. Terpenes, including 1,8-cineole, have been shown to have both antibiotic properties and the ability to block receptors that receive signals from various autoinducers [61]. The inhibition of the QS pathway is of great biological importance and undoubtedly contributes to the antibacterial activity of eucalyptol.

It is interesting that the QS inhibitory compounds described so far influenced only the virulence of bacteria, but not their basic vital functions [62]. However, the research of Li et al. [63] showed that 1,8-cineole changed the shape and size of the bacterial cell (for both Gram-negative and Gram-positive bacteria). In addition, bacterial cells treated with this compound underwent apoptosis (*S. aureus*), because they showed a strong condensation of nuclear chromatin located in the central part of the nucleoplasm and necrosis (*E. coli*), in which there was a clear reduction of nucleoplasm and nuclear chromatin accumulated in the nuclear envelope. The studies of these authors have shown that cineole has a better effect against *E. coli* because, unlike *S. aureus*, their cells had ruptured the cell wall and membrane. This fact is of great clinical importance because, due to the easy spread of virulence factors among Gram-negative bacteria and the limited action of available antibiotics in this situation, it becomes desirable to search for such compounds of natural origin that will cover this group of microorganisms in their spectrum of activity. 1,8-Cineole owes its antimicrobial activity to its hydrophobicity, making bacteria with an outer lipopolysaccharide membrane susceptible to this compound.

Şimşek et al. [55] showed that 1,8-cineole can enhance the antimicrobial effect of antiseptics, which complements the results of the studies by Li et al. [63]. In order to show a synergistic effect, both compounds must reveal the potentialism that occurs when their effect is identical, but at the same time, each of them has a different mechanism of action (acts on a different part of the cell) and recognizes a different receptor. Chlorhexidine is known to damage the bacterial cell wall, thereby increasing its permeability, which suggests that cineole acts on an already disturbed bacterial cell and somehow inactivates cellular components, as confirmed by the studies of Yu et al. [58] However, based on the research by Li et al. [63], where the disruption of the cell wall was visualized in *E. coli* after the action of 1,8-cineole, it can be presumed that cineole in combination with chlorhexidine would also be able to show an additive effect, i.e., one where both compounds have the same mechanism of action and recognize the same (or very similar) receptor. In this respect, cineole is a very interesting compound of natural origin and undoubtedly worth the attention of microbiologists.

## 4. Anti-Inflammatory Activity

Extensive inflammation is a serious problem in infectious diseases. Admittedly, the controlled accumulation of inflammatory factors, such as prostaglandins, leukotrienes, TNF-α, or interleukins (e.g., IL-1, IL-6, IL-8, IL-10), has a beneficial effect in the fight against microorganisms. However, in principle, a favorable pro-inflammatory response through overstimulation may contribute to the development of dangerous syndromes such as sepsis and septic shock [64]. Therefore, it is important that the antimicrobial drug used also has anti-inflammatory activity. Eucalyptol has been used in studies of inflammation in rats, i.e., carrageenan paw edema and cotton granule granuloma. In the case of carrageenan paw edema in rats, 1,8-cineole at doses of 100, 200, and 400 mg/kg caused a significant reduction in paw swelling by 26%, 26%, and 46%, respectively. In the case of the granuloma induced by cotton granules, after 7 days in the group that was administered eucalyptol at a dose of 400 mg/kg, the inhibition of the wet and dry mass of the granuloma was 37 and 40%, respectively [65].

1,8-Cineole can be used to treat rhinitis and sinusitis. A total of 150 people aged 18–57 were qualified for the study, two-thirds of whom were women. The test subjects received 1,8-cineole or a placebo. After 7 days of therapy in the 1,8-cineole group, only six people (8%) showed no 50% improvement. On the other hand, in the placebo group, such a condition was observed in 55 people (73%). The clear differences between the two groups indicate that the use of 1,8-cineole significantly improves health in people with rhinitis and sinusitis and allows avoiding antibiotic therapy [66].

Eucalyptol, as a compound having mucolytic, bronchodilatory, and anti-inflammatory properties, is also used in the treatment of bronchitis. A total of 242 people aged 18–70 years old, without any additional illnesses, and with bronchitis lasting no longer than 7 days, were qualified for the study. Half of the patients received cineole in three doses of 200 mg daily within 10 days, and the rest received a placebo. After four days of therapy, a significant reduction in the frequency of coughing was observed in those receiving 1,8-cineole [67].

It was already indicated in 1998 that 1,8-cineole inhibits the synthesis of leukotriene B_4_ and prostaglandin E_2_ [68] and reduces the production of pro-inflammatory factors, such as TNF-α, IL-1β, IL-6, and IL-8 in monocytes [69] and TNF-α, IL-1β, IL-4, and IL-5 in lymphocytes, by over 60% [28]. A 2017 study using in vitro cultured human monocytes that were collected from healthy, non-smoking subjects showed a dose-dependent inhibition of pro-inflammatory factors, such as IL6, IL1β, IL-8, and TNFα, at systemic 1,8-cineole concentrations in the range of 0.15–1.5 µM. The partial inhibition (20–40%) of IL-1β and IL-6 occurred at an exhaled eucalyptol concentration of 0.3 µM. These data demonstrated for the first time the dose-dependent anti-inflammatory activity of 1,8-cineole at clinically relevant systemic and expired air concentrations, as, in lipopolysaccharide (LPS) stimulated human monocytes, 1,8-cineole showed the complete inhibition of IL -6 at plasma concentrations of 0.6 µM [70,71].

LPS occurs on the surface of microorganisms, but is a foreign substance to higher organisms. LPS facilitates the colonization of host cells by bacteria through adhesion and induces a rapid response of the immune system. It interacts with host cells, e.g., monocytes, macrophages, polynuclear leukocytes, B and T lymphocytes, and various endothelial and epithelial cells. One of the pathways by which LPS triggers inflammation is where the adapter proteins involved in LPS-derived signal transduction are TRAM and TRIF. This pathway leads to the NF-κB-activated transcription of genes responsible for the production of proteins related to the body’s immune response [72].

A study on human cell lines using LPS as an inflammation stimulant showed that eucalyptol extract at a concentration of 0.6 mg/μL was able to reduce inflammation by suppressing p65—the NF-κB promoter [73].

In addition, eucalyptol is able to lower the level of pattern recognition receptors involved in LPS signaling, leading to a reduction in the phosphorylation of downstream transcription factors NF-κB and p38 [74].

## 5. Therapeutic Uses

1,8-Cineole, due to its anti-inflammatory, mucolytic, antiseptic, and antimicrobial properties, is used as a component of many drugs used in various diseases.

1,8-Cineole affects the activity of cilia in the respiratory epithelium, improves the transport of secretions, helps to clear the respiratory tract, and facilitates expectoration. Moreover, by showing mucolytic and anti-inflammatory effects, it reduces the severity of symptoms of acute sinusitis, i.e., headache, sensitivity of the trigeminal nerve to pressure, stuffy nose, and the excessive discharge of secretions.

By irritating the mucous membranes of the upper respiratory tract and stomach, 1,8-cineole dilates the bronchi, stimulates their secretory activity, and facilitates the expectoration of the remaining secretions.

1,8-cineole is also an ingredient in rhinitis medications. It helps to cleanse the nasal cavities from secretions, and helps to restore the proper functions of the mucous membranes. In addition, it soothes irritations and cleanses, soothes, and protects the epidermis and mucous membranes. 1,8-Cineole has also been used in diseases of the biliary tract, as it has a choleretic effect, preventing bile stasis and diastolic and reducing pain in spasms [67,75].

Table 3 shows examples of products approved in medical treatment in Poland that contain 1,8-cineole.

## 6. Eucalyptol Metabolism

Eucalyptol is rapidly absorbed and can be detected in the blood 5 min after inhalation, although the maximum plasma concentration is reached approximately 18 min after inhalation [76,77,78].

After oral ingestion in gastro-resistant capsules, eucalyptol is absorbed in the small intestine and then metabolized in the liver, where it is acted upon by cytochrome P450 enzymes (CYP). The main metabolites include 2-α-hydroxy- and 3-α-hydroxy-1,8-cineole, whose compounds are then conjugated with glucuronic acid and excreted in the urine [79,80]. Miyazawa et al. [81] determined that, in humans, the major CYP isoform responsible for the formation of 2-α-hydroxy-1,8-cineole is CYP3A4. In vitro biotransformation studies of eucalyptol were also performed using recombinant CYP3A4 and CYP3A5 co-expressed with human CYP reductase in *E. coli* cells. Then, more 3-α-hydroxy-1,8-cineole was obtained [80]. The metabolism of 1,8-cineole after the consumption of sage tea was also investigated. In addition to 2-α-hydroxy- and 3-α-hydroxy-1,8-cineole, the presence of 7-hydroxy-1,8-cineole and 9-hydroxy-1,8-cineole was also detected. Studies have also been carried out with radiolabeled eucalyptol using [^2^H_3_]-1,8-cineole, [9/10-^2^H_3_]-2-hydroxy-1,8-cineole, and [^13^C, ^2^H_2_]-9-hydroxy-1,8-cineole as internal standards. The dominant metabolite was 2-hydroxy-1,8-cineole, with a maximum plasma concentration of 86 nmol/L. 1,8-Cineole alone showed a low peak plasma concentration of 19 nmol/L.

It is worth emphasizing that eucalyptol may affect the plasma concentration of other drugs [82]. In studies in rats, eucalyptol or a placebo was sprayed for 5–10 min for four days. Brain concentrations of aminopyrine, amphetamine, zoxazolamine, and pentobarbital were found to be significantly reduced within 2–6 h after drug administration, compared to the controls. In five healthy human volunteers who received aerosol eucalyptol for 10 min for 10 consecutive days, an increase in the plasma clearance of aminopyrine was observed in four of the five participants. All drugs were administered 24 h after the last aerosolization, and the authors concluded that eucalyptol is likely to be an inducer of CYP enzymes in the liver, even when administered by inhalation [78].

Studies were also carried out on the microsomal fraction obtained from the liver of female Wistar rats that had been pretreated with phenobarbital. Eucalyptol inhibited the activity of CYP2B1, which suggests that it may affect the metabolism of xenobiotics, which are substrates for this isoenzyme [83]. Importantly, CYP2B1 is homologous to human CYP2B6 [84]. This CYP isoform is involved in the metabolism of many compounds, including clinically used drugs (e.g., cyclophosphamide (CPA), calcium channel antagonists, 3-hydroxy-3-methyl-glutaryl-CoA(HMGCoA) reductase inhibitors, and thiazolidinediones), endogenous and environmental chemicals, or pesticides [85].

In contrast, in a more recent in vitro study of the effect of 1,8-cineole on CYP levels, a dose-dependent decrease in the following CYP isoforms was observed: 1A2, 2C8, 2C9, 2C19, and 3A4, especially at compound concentrations of 100 and 500 μg/mL [86]. Therefore, further studies on the possible drug interactions between eucalyptol and the induction or inhibition of CYP seems necessary [78].

The urinary excretion of eucalyptol was also found within 10 h when examining the metabolism of eucalyptol. The main metabolite was 2-hydroxycineole at 20.9%. Further compounds observed in urine were 9-hydroxycineole (17.2%), 3-hydroxycineole (10.6%), and 7-hydroxycineole (3.8%) [87]. Eucalyptol is also excreted in the exhaled air, which means that it is able to reach the lungs, peripheral airways, and sinuses when ingested orally [78].

Eucalyptol biotransformation metabolites are also excreted in breast milk. Eucalyptol in a single oral dose corresponding to one Soledum capsule (100 mg) (Klosterfrau Healthcare, Cologne, Germany) was administered orally to nursing mothers. In small amounts of milk, the presence of as many as ten metabolites was detected, including 2-oxo- and 3-oxo-1,8-cineole, 2α- and 2β-hydroxy-1,8-cineole, 3α-hydroxy-1,8-cineole, 4-, 7-, and 9-hydroxy-1,8-cineoles, 2,3-dehydro-1,8-cineole, and 2,3-α-epoxy-1,8-cineoles [88,89].

## 7. Eucalyptol Biotransformation

Antibiotics commonly used in the treatment of infections, which get into surface waters and soil, negatively affect the local ecosystems. For this reason, it is important to identify possible degradation pathways for any new antimicrobial drug, including 1,8-cineole. The first stage of degradation is often biotransformation processes.

The first attempts to biotransform 1,8-cineole in microbial cultures were reported in 1979, when MacRae et al. [90] used the *Pseudomonas flava* UQM 1742 strain, which had been isolated from the surface of eucalyptus leaves, to transform this compound. The microorganism grew on the medium with the addition of 1,8-cineole at the concentration of 0.5 g/L. The substrate conversion was 20% after 20 h of transformation and the following three products were obtained: (1*S*)-2α-hydroxy-1,8-cineole, (1*S*)-2β-hydroxy-1,8-cineole, and (1*S*)-2-oxo-1,8-cineole. In the *Bacillus cereus* culture (UI-1477), (1*R*)-6α-hydroxy-1,8-cineole was obtained with a yield of 74% [91]. In turn, Williams et al. [92] isolated a strain of *Rhodococcus* sp. C1 capable of growing in a medium with 1,8-cineole as the sole carbon source. During the exponential growth phase, (1*R*)-6β-hydroxy-1,8-cineole and 6-oxo-1,8-cineole were formed transiently. The lactone, 5,5-dimethyl-4-(3′-oxobutyl)-4,5-dihydrofuran-2(3H)-one, was also isolated from the reaction mixture. This compound is not a direct oxidation product, but is the result of the non-enzymatic lactonization of the 3-(1-hydroxy-l-methylethyl)-6-oxoheptanoic acid intermediate during extraction procedures.

Rasmussen et al. [93] described the isolation of 44 microorganisms from activated sludge that were able to grow in the presence of 1,8-cineole as the sole carbon source. From the isolated strains of *Novosphingobium subterranea*, it allowed to obtain five compounds, including 2α- and 2β-hydroxy-1,8-cineole, and the 2-oxo derivative of 1,8-cineole. From the same pellet at pH = 2, a strain of the fungus *Penicillium purpurogenum* was isolated, which was capable of converting 1,8-cineole into 3-hydroxy-1,8-cineole and 3-oxo-1,8-cineole [93].

Biotransformations of 1,8-cineole were also performed in *Mucor ramannianus* and *Aspergillus niger* cultures. In both cultures, the main products observed were 2-exo-hydroxy-1,8-cineole and 3-exo-hydroxy-1,8-cineole, at a substrate concentration of 1 g/L after 24 h. As the substrate concentration was raised to 5 g/L, the substrate conversion decreased from 99% to 21.7% in the case of *M. ramannianus* after 5 days of transformation. The second strain was unable to hydroxylate 1,8-cineole at such a high substrate concentration [94].

An interesting example confirming that hydroxylation and oxidation are the first steps in the biodegradation of 1,8-cineole is the biotransformation using *Pleurotus ostreatus* ICFC 153/99 and *Favolus tenuiculus* ICFC 383/00 cultures. The strains were grown on solid-state fermentation (SSF) containing 350 g of wet spent leaf waste of *E. cinerea*. The transformation products were 1,3,3-trimethyl-2-oxabicyclo[2.2.2]octane-6-ol and 1,3,3-trimethyl-2-oxabicyclo[2.2.2]octane-6-one [95].

Extensive screening studies on the ability to transform 1,8-cineole were carried out by Abraham [96], who used 100 strains (40 bacterial strains and 60 fungal strains) from the collection of the Gesellschaft für Biotechnologische Forschung, Braunschweig, Germany. Of the strains tested, only 20% of bacteria (Gram-positive and Gram-negative) compared to 62.5% of fungi (deuteromycetes, ascomycetes, and basidiomycetes) was capable of forming 2α-hydroxy-1,8-cineole from 1,8-cineole. On the other hand, the 3α-hydroxy derivative of 1,8-cineole was formed by 17% of bacterial strains and 32.5% of fungal strains [80,96].

P450_cin_ (CYP176A1) is responsible for the regio- and stereospecific hydroxylation of 1,8-cineole to (1*R*)-6β-hydroxycineole. This enzyme was first isolated from *Citrobacter braakii*—a microorganism that grew in the 1,8-cineole medium. The hydroxylation reaction is therefore considered to be the first step in the biodegradation of cineole, as it determines the survival of the microorganism on the medium with this compound [97]. CYP176A1 is a close analogue of CYP101A1 from *Pseudomonas putida*—one of the best known bacterial CYPs, which is highly effective in catalyzing the camphor hydroxylation reaction and is involved in the degradation of this compound [98]. Similar roles are played by the related camphor hydroxylases CYP101D1 and CYP101D2 that are isolated from *Novosphingobium aromaticivorans* DSM 12444, as well as CYP108A1 from *Pseudomonas* sp., which is capable of α-terpineol hydroxylation [99].

The second step in the microbial degradation of eucalyptol is the oxidation of (1*R*)-6β-hydroxycineole to (1*S*)-6-ketocineole, a reaction catalyzed by 6β-hydroxycineole dehydrogenase. It is noteworthy that, of the four possible isomers of 6-hydroxycineole, only (1*R*)-6β-hydroxycineole is oxidized. The next step in the degradation of 1,8-cineole is enzymatic Baeyer–Villiger oxidation, which converts the cyclohexanone ring into a lactone which is then hydrolyzed and can be rearranged by the elimination of water to form a tetrahydropyranyl derivative. The remaining steps in biodegradation are probably the classic cleavage of acetate units by β-oxidation, which explains the possible use of 1,8-cineole as a sole carbon source by a number of bacterial strains [80].

*Escherichia coli* cells with cloned CYP176A1 are used in in vitro studies. The enzyme then uses two redox partners in order to catalyze: cindoxin (Cdx) containing FMN and *E. coli* flavodoxin reductase, which in turn is dependent on NADPH [100]. In a 3.5 L culture of *E. coli* expressing the plasmid containing CYP176A1 with its redox partners, 3.1 g (26%) of high optical purity (1*R*)-6β-hydroxycineole was produced after incubating the culture for 3 days with eucalyptol [37]. It is worth noting that, in such a system, CYP176A1 is able to catalyze the less selective oxidation of other monoterpenes as well, such as both camphor enantiomers [101].

CYP176A1 was found to contain an asparagine residue (N242) at a key site, where most other CYPs have threonine. The CYPs use this conserved threonine residue in order to provide the protonation of the distal hydroperoxide oxygen, since the protonation of the proximal oxygen leads to the formation of hydrogen peroxide (the disconnection of electron consumption without product formation). However, studies have shown that asparagine in CYP176A1 is not a functional threonine replacement but controls the stereo- and regioselectivity of oxidation [102]. The fact that CYP176A1 is catalytically competent in 1,8-cineole hydroxylation (its rate of NADH consumption is 950 min^−1^ and coupling ~80%) drew the attention of several research groups and inspired a detailed analysis of its structure and mechanistic issues [103]. It was found that the mutant N242A of this enzyme (the Asn242 residue replaced with alanine) catalyzes the selective oxidation of 1,8-cineole to (1*S*)-6α-hydroxycineole (90% of all products produced), and not to (1*R*)-6β-hydroxycineole, i.e., the enantiomer that is observed when used with a wild-type enzyme [104]. After replacing asparagine with threonine (N242T), the product profile changed further, resulting in three hydroxycineole isomers: (1*R*)-6β-hydroxycineole (47%), (1*S*)-6α-hydroxycineole (22%), and (1*R*)-6α-hydroxycineole (31%) [100,103].

**Figure 2:**
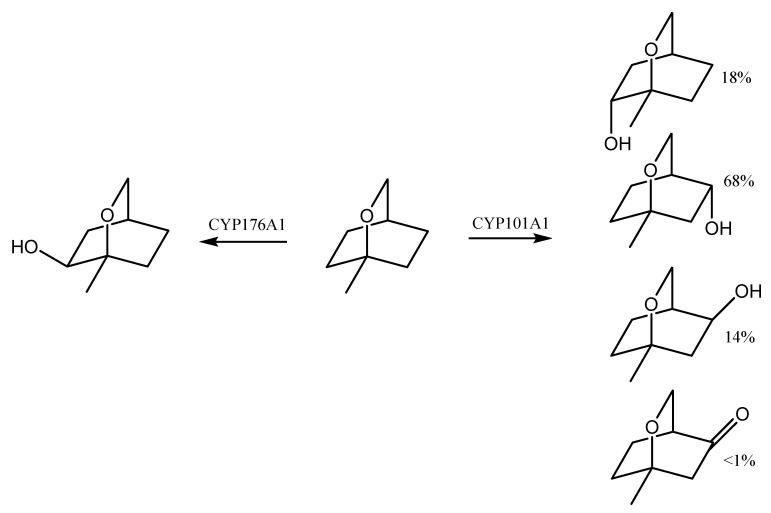
Biotransformation of 1,8-cineole by CYP176A1 and CYP101A1 [105].

The above studies of the effect of point mutations on the stereoselectivity of 1,8-cineole biotransformation show that it is the hydrogen bond between the Asn242 residue and the 1,8-cineole ether oxygen, and not the hydrophobic interactions, that plays a key role in guiding the P450 oxidation regiochemistry (Figure 2). Moreover, in the absence of any hydrogen bond formation, the relative reactivity of the various oxidation sites can determine the regioselectivity of the reaction [104].

Eucalyptol can also be hydroxylated by CYP101A1 from *P. putida*. This enzyme was able to oxidize 1,8-cineole into three major products: (1*S*)-6α-hydroxycineole and 5α- and 5β-hydroxycineole, in the ratio 18:68:14. The formation of a trace amount of 5-keto-1,8-cineole was also observed. Therefore, a number of CYP101A1 variants capable of hydroxylating 1,8-cineole at the α-position were constructed. For example, the F87V/Y96F/L244A (VFA) variant showed a strong preference for 6α-oxidation (90%), yielding optically pure (1*S*)-6α-hydroxy-1,8-cineole. In turn, the variant F87W/Y96F/L244A/V247A (WFAL) made it possible to obtain 5α-hydroxy-1,8-cineole as the main product (88%, ee = 86% (1*S*)). Additionally, 10% of 6α-hydroxy-1,8-cineole and 2% of 5β-hydroxy-1,8-cineole were formed [105].

A similar stereoselectivity was displayed by the engineered CYP102A1 mutants. The CYP102A1 mutant RLYFGVQ generated 6α-hydroxy-1,8-cineole as the sole product (>98%). The RLYFAIP variant, on the other hand, was less selective for the oxidation of 1,8-cineol, generating significant amounts of 5α-hydroxy-1,8-cineole in addition to the main product, 6α-hydroxy-1,8-cineole [105].

**Figure 3:**
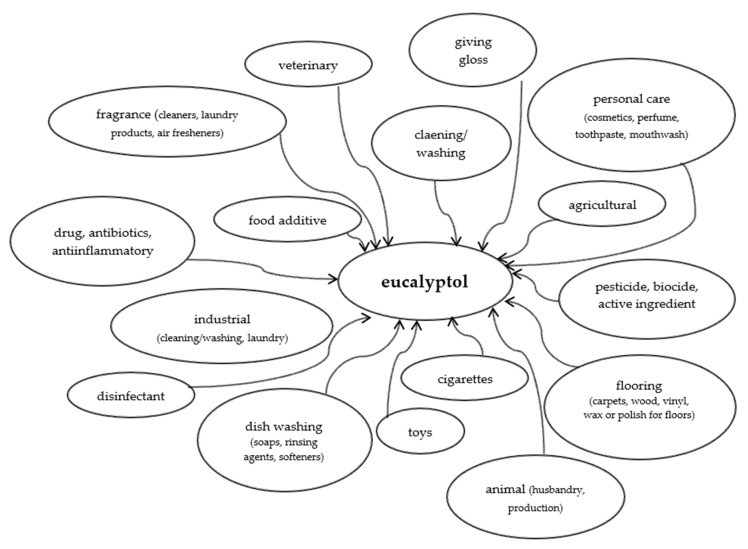
Spheres of wide use of eucalyptol (based on [106]).

Figure 3 shows how ubiquitous is eucalyptol. There is probably no sphere of our life in which it would not be used.

## 8. Conclusions

One should consider whether antibiotics really are the final form of therapy in bacterial infections. Perhaps, in the early stages of the disease, the use of therapies supported by natural compounds, such as cineole, should be considered. Choosing this path will often allow obtaining the desired therapeutic result, limiting the possibility of bacteria acquiring additional resistance mechanisms. Of course, in the case of a rapidly progressive infection or a life-threatening infection, the most appropriate action that should be taken is the rapid administration of an appropriately selected antibiotic. However, also in this case, a combination therapy can be considered, which will protect, at least partially, against the emergence or growth of a resistance to antibiotics of “last resort” in primary sensitive microorganisms.

## Figures and Tables

**Table 1:** Plant resources of 1,8-cineole.

Plant	Percentage [%]	Ref.
*Eucalyptuskochii* subsp. *borealis*	97.32	[10]
*Eucalyptus kochii* subsp. *plenissima*	96.55	[10]
*Eucalyptus globulus Labill.*	*95.13*	[11]
*Eucalyptuskochii* subsp. *plenissima*	92.31	[12]
*Eucalyptus* *horistes*	90.17	[12]
*Rosmarinus officinalis* L.	88.9	[13]
*Eucalyptus alba*	88.1–76.5	[14]
*Eucalyptus globulus* Labill.	87.82	[15]
*Eucalyptus cinerea*	87.3	[16]
*Eucalyptus baueriana* F. Muell	87.1	[16]
*Eucalyptus* *polybractea*	87.32	[12]
*Eucalyptus smithii* R. Bake	86.4	[17]
*Eucalyptus leucoxylon* var. *rostellata* Miq	85.5	[18]
*Eucalyptus cinere*	85.32	[19]
*Eucalyptus cinere*	84.4	[20]
*Eucalyptus smithii* R. Bake	84.27	[21]
*Salvia fruticosa*	83.7	[22]
*Eucalyptus smithii* R. Baker	83,2	[16]
*Eucalyptus polybractea*	82.95	[10]
*Eucalyptus bridgesiana* R. T. Baker	82.6	[16]
*Eucalyptus radiata* Sieb. ex DC ssp radiata	80.8	[17]
*Nepeta italica* L	80.8	[23]
*Eucalyptus intertexta* var. *intertexta* R.T. Baker	80,6	[18]
*Eucalyptus microtheca F. Muell.*	80.3	[16]
*Eucalyptus globulus* Labill.	78.9	[24]
*Eucalyptus* *loxophleba*	78.78	[10]
*Ocimum canum* Sims.	78.3	[25]
*Eucalyptus globulus*	77.02	[10]
*Eucalyptus foecunda Schau.*	75.5	[16]
*Eucalyptus sargentii* subsp. Sargantii Maiden	75.5	[18]
*Eucalyptus pulverulenta* Sims;	75.1	[16]
*Laurus nobilis* L.	73	[26]
*Eucalyptus globulus* Labill.	72.8	[27]
*Eucalyptus globulus* Labill.	72.5	[28]
*Eucalyptus spathulata*	72.5	[29]
*Eucalyptus globulus* Labill.	71.3	[30]
*Amomum subulatum* Roxb.	71.27	[31]
*Eucalyptus globulus* Labill.	71.2	[21]
*Eucalyptus* *cinerea*	70.7	[32]
*Thymus mastichina*	70.60–52.01	[33]
*Eucalyptus cinere*	70.4	[34]
*Eucalyptus camaldulensis* Dehn., var. mysore,	70.4	[35]
*Eucalyptus globulus* Labill.,	70.1	[17]
*Melaleuca viridiflora* Soland. ex Gaertn.,	70	[36]
*Rosmarinus officinalis* L.	69.33	[37,38]
*Eucalyptus* sideroxylon	69.2	[32,34]
*Eucalyptus resinifera* Smith	68	[39]
*Eucalyptus bicostata*	68.0	[32,34]
*Eucalyptus* *loxophleba*	66.93	[10]
*Eucalyptus propinqua* Deane and Maiden	67.5	[16]
*Artemisia afra* Willd.	67.37	[40]
*Eucalyptus* *loxophleba*	66.93	[10]
*Eucalyptus torquata*	66.9	[29]
*Callistemon citrinus* (Curtis) Sheels (*C. lanceolatus* DC.)	66.3	[41]
*Eucalyptus globulus* Labill.	66.1	[42]
*Cinnamomum glanduliferum* Bark	65.87	[43]
*Eucalyptus globulus* Labill.	64.5	[44]
*Melaleuca alternifolia* (Maiden et Betche) Cheel	64.1	[45]
*Eucalyptus globulus* Labill.	63.8	[46,47]
*Eucalyptus longifolia* Link & Otto	63.3	[48]
*Eucalyptus globulus* Labill.	62.5	[49]
*Myrtus communis* L.	61.5	[50]
*Nepeta sulfurifloral* P.H. David	61.5	[23]
*Lavandula stoechas* L.	61.36	[50]
*Amomum subulatum* Roxb.	61.3	[51]
*Cinnamomum camphora* (L.) Nees et Ebermaier	60.7	[36]
*Amomum subulatum* Roxb.	60	[52]
*Eucalyptus maidenii* F. Muell.	60	[46]
*Eucalyptus globulus* Labill.	60	[53,54]

**Table 2:** Antibacterial activity of 1,8-cineole depending on its content in various plants.

Essential Oil	Inhibition of Bacterial Growth (the Largest > the Smallest)	Reference
*Eucalyptus alba*(1,8-cineole: **76.5–88.1%**)	*S. aureus* ATCC 29213 > *E. coli* ATCC 25922 = *E. faecalis* ATCC 29212 > *P. aeruginosa* ATCC 27853	[14]
*Eucalyptus cinerea*(1,8-cineole: **70.7%**)	*S. aureus* ATCC 25932 > *E. coli* ATCC 25922 > *P. aeruginosa* ATCC 227853 > *E. faecalis* ATCC 292112	[32]
*Eucalyptus sideroxylon*1,8-cineole: **69.3%**)	*S. aureus* ATCC 25932 > *E. coli* ATCC 25922 > *P. aeruginosa* ATCC 227853 > *E. faecalis* ATCC 292112	[32]
*Eucalyptus bicostata*(1,8-cineole: **68%**)	*S. aureus* ATCC 25932 > *E. faecalis* ATCC 292112 > *E. coli* ATCC 25922 > *P. aeruginosa* ATCC 227853	[32]
*Eucalyptusfasciculosa*(1,8-cineole: **55%**)	*S. aureus* ATCC 25932 > *E. coli* ATCC 25922 > *E. faecalis* ATCC 292112	[32]
*Eucalyptusmacarthurii* (1,8-cineole: **55%**)	*S. aureus* ATCC 25932 > *E. faecalis* ATCC 292112 > *E. coli* ATCC 25922 = *P. aeruginosa* ATCC 227853	[32]
*Eucalyptuscitriodora* (1,8-cineole: **54%**)	*E. coli* ATCC 25922 > *P. aeruginosa* ATCC 227853 > *E. faecalis* ATCC 292112 = *S. aureus* ATCC 25932	[32]
*Eucalyptus kochii* subsp. *borealis*(1,8-cineole: **97.32%**)	*E. coli* ATCC 25922 = *S. aureus* ATCC 29213 > *S. aureus* MRSA NCTC 10442 = *E. faecalis* ATCC VRE 51299 = *S.* Typhimurium ATCC 13311 = *A. baumannii* NCTC 7844 > *S. epidermidis* NCTC 11047 = *C. albicans* ATCC 90028 > *E. faecalis* ATCC 29212 > *P. aeruginosa* ATCC 27853	[10]
*Eucalyptus kochii* subsp. *plenissima plenissima*(1,8-cineole: **96.55%**)	*S. aureus* ATCC 29213 = *S.* Typhimurium ATCC 13311 = *E. coli* ATCC 25922 = *A. baumannii* NCTC 7844 > *S. aureus* MRSA NCTC 10442 > *C. albicans* ATCC 90028 > *E. faecalis* ATCC 29212 = *E. faecalis* VRE ATCC 51299 = *S. epidermidis* NCTC 11047 = *P. aeruginosa* ATCC 27853	[10]
*Eucalyptus polybractea*(1,8-cineole: **82.95%**)	*E. faecalis* VRE ATCC 51299 = *S. epidermidis* NCTC 11047 = *A. baumannii* NCTC 7844 > *S. aureus* MRSA NCTC 10422 = *E. coli* ATCC 25922 = *P. aeruginosa* ATCC 27853 > *S. aureus* ATCC 29213 = *E. faecalis* ATCC 29212 = *C. albicans* ATCC 90028 = *S.* Typhimurium ATCC 13311	[10]
*Eucalyptus loxophleba 2*(1,8-cineole: **78.78%**)	*A. baumannii* NCTC 7844 > *S.* Typhimurium ATCC 13311 > *S. aureus* ATCC 29213 = S. *aureu*s MRSA NCTC 10442 = *E. faecalis* VRE ATCC 51299 > *S. epidermidis* NCTC 11047 = *C. albicans* ATCC 90028 = *E. coli* ATCC 25922 > *E. faecalis* ATCC 29212 > *P. aeruginosa* ATCC 27853	[10]
*Eucalyptus globulus*(1,8-cineole: **77.02%**)	*S.* Typhimurium ATCC 13311 > *S. aureus* MRSA NCTC 10442 = *A. baumannii* NCTC 7844 > *S. aureus* ATCC 29213 = *E. faecalis* VRE ATCC 51299 = *S. epidermidis* NCTC 11047 > *E. coli* ATCC 25922 > *P. aeruginosa* ATCC 27853 > *E. faecalis* ATCC 29212 = *C. albicans* ATCC 90028	[10]
*Eucalyptus loxophleba 1*(1,8-cineole: **66.93%**)	*E. faecalis* VRE ATCC 51299 = *A. baumannii* NCTC 7844 > *P.aeruginosa* ATCC 27853 > *S. aureus* MRSA NCTC 10442 = *S.* Typhimurium ATCC 13311 = *E. coli* ATCC 25922 > *S. aureus* ATCC 29213 = *E. faecalis* ATCC 29212 = *S. epidermidis* NCTC 11047 = *C. albicans* ATCC 90028	[10]

**Table 3:** Medicinal preparations containing 1,8-cineole.

Product (Polish Name)	1,8-Cineole Content	Application
SOLEDUM FORTE	pure 1,8-cineole (100%)	non-suppurative sinusitis
ROWATINEX	3 mg/capsule	antibacterial (stuffy nose)
ROWACHOL	2 mg/capsule	gallstones, diseases of the biliary tract
TERPICHOL PLUS	1.9 mg/capsule	faciliting digestion (proper production of bile)
SALVIASEPT	0.6 g/100 g	inflammation of the oral cavity
OTRIVIN MENTHOL 0.1%	auxiliary substance	rhinitis
AFRIN ND MENTHOL	auxiliary substance	rhinitis
VICKS SINEX ALOE AND EUCALYPTUS	auxiliary substance	rhinitis
RINOZINE	auxiliary substance	rhinitis, sinusitis

## Data Availability

Publicly available datasets were analyzed in this study. This data can be found here: https://essentialoils.org/ (accessed on 8 August 2021) and https://www.ncbi.nlm.nih.gov/ (accessed on 8 August 2021).

## Sample Availability

Samples of the compounds are not available from the authors.
